# Comprehensive Analysis of Prognostic and Genetic Signatures for General Transcription Factor III (GTF3) in Clinical Colorectal Cancer Patients Using Bioinformatics Approaches

**DOI:** 10.3390/cimb43010002

**Published:** 2021-04-27

**Authors:** Gangga Anuraga, Wan-Chun Tang, Nam Nhut Phan, Hoang Dang Khoa Ta, Yen-Hsi Liu, Yung-Fu Wu, Kuen-Haur Lee, Chih-Yang Wang

**Affiliations:** 1 Ph.D. Program for Cancer Molecular Biology and Drug Discovery, College of Medical Science and Technology, Taipei Medical University and Academia Sinica, Taipei 11031, Taiwan; g.anuraga@unipasby.ac.id (G.A.); d621109004@tmu.edu.tw (H.D.K.T.); 2 Graduate Institute of Cancer Biology and Drug Discovery, College of Medical Science and Technology, Taipei Medical University, Taipei 11031, Taiwan; yeas0310@hotmail.com; 3 Department of Statistics, Faculty of Science and Technology, Universitas PGRI Adi Buana, Surabaya, East Java 60234, Indonesia; 4 NTT Institute of Hi-Technology, Nguyen Tat Thanh University, Ho Chi Minh City 700000, Vietnam; pnnam@ntt.edu.vn; 5 School of Chinese Medicine for Post-Baccalaureate, I-Shou University, Kaohsiung 82445, Taiwan; isu10556037a@cloud.isu.edu.tw; 6 Department of Medical Research, Tri-Service General Hospital, School of Medicine, National Defense Medical Center, Taipei 11490, Taiwan; qrince@yahoo.com.tw; 7 Cancer Center, Wan Fang Hospital, Taipei Medical University, Taipei 11031, Taiwan

**Keywords:** colorectal cancer, bioinformatics, GTF3A, GTF3B, GTF3C1, GTF3C2

## Abstract

Colorectal cancer (CRC) has the fourth-highest incidence of all cancer types, and its incidence has steadily increased in the last decade. The general transcription factor III (GTF3) family, comprising GTF3A, GTF3B, GTF3C1, and GTFC2, were stated to be linked with the expansion of different types of cancers; however, their messenger (m)RNA expressions and prognostic values in colorectal cancer need to be further investigated. To study the transcriptomic expression levels of GTF3 gene members in colorectal cancer in both cancerous tissues and cell lines, we first performed high-throughput screening using the Oncomine, GEPIA, and CCLE databases. We then applied the Prognoscan database to query correlations of their mRNA expressions with the disease-specific survival (DSS), overall survival (OS), and disease-free survival (DFS) status of the colorectal cancer patient. Furthermore, proteomics expressions of GTF3 family members in clinical colorectal cancer specimens were also examined using the Human Protein Atlas. Finally, genomic alterations of GTF3 family gene expressions in colorectal cancer and their signal transduction pathways were studied using cBioPortal, ClueGO, CluePedia, and MetaCore platform. Our findings revealed that GTF3 family members’ expressions were significantly correlated with the cell cycle, oxidative stress, WNT/β-catenin signaling, Rho GTPases, and G-protein-coupled receptors (GPCRs). Clinically, high GTF3A and GTF3B expressions were significantly correlated with poor prognoses in colorectal cancer patients. Collectively, our study declares that GTF3A was overexpressed in cancer tissues and cell lines, particularly colorectal cancer, and it could possibly step in as a potential prognostic biomarker.

## 1. Introduction

According to global cancer statistics, colorectal cancer (CRC) causes more than 700,000 deaths every year, and there will be an estimated 53,200 CRC deaths in United States in 2020 [1,2]. This evidence makes CRC one of the deadliest cancer types, along with lung cancer, liver cancer, and stomach cancer. The incidence has slightly increased year to year as globalization trends have grown in developing countries [3]. More people are shifting to western diets and lifestyles; therefore, CRC nowadays is known as a disease of modernity [4]. Furthermore, the number of cancer patients is increasing due to population growth and aging [5]. Recently, researchers demonstrated rapid advances in the field of cancer therapy, where genetic alterations and the dysfunction of signal transduction pathways play important roles in cancer development [6,7,8]. Although cancer development’s underlying mechanisms have been extensively studied, CRC patients in particular are still coping with low survival rates. Therefore, novel and effective therapeutic treatments and drug development are crucial [9,10,11].

However, a lack of information on biological pathways has been a problem for years. Fortunately, with the rapid development of big data and more-rapid computing abilities, several huge publicly available datasets have been established for academic research purposes. In biomedical studies, bioinformatics analyses play a critical part due to their strength and rapidness in screening potential candidates for particular diseases [12,13,14,15,16,17]. Various public databases, including the Gene Expression Omnibus (GEO), which includes more than 94,000 datasets and over 2 million samples, are widely employed in cancer research for both raw and processed dataset analyses [18]. Meanwhile, since altered gene expressions of both oncogenic and tumor-suppressive genes have long been proposed to play a part in cancer development, we followed this approach to explore biomarker prognosticators by examining several databases. Therefore, the Oncomine platform has been used to query transcript expression profiles of numerous cancer types and subtypes, and it offers multiple types of analyses, allowing researchers to compare variations in gene expressions between tumorous and normal matched tissues [19,20,21,22].

Members of the general transcription factor III (GTF3) family function as RNA polymerase III transcription factors to induce transcription of 5S ribosomal (r)RNA genes, which are involved in ribosomal large-subunit biogenesis. Currently, this family of transcription factors has four members, named in order of when they were discovered: GTF3A, GTF3B, GTF3C1, GTF3C2. Northern analyses revealed their expressions in several tissues examined; e.g., fluorescent in situ hybridization was used to locate the human TFIIIA gene (GTF3A) on chromosome band 13q12.3→q13.1 [23]. A previous study demonstrated that GTF3C1 is an immune related marker for triple-negative breast cancer (TNBC) [24], but the supporting evidence in CRC is inconclusive. So far, however, no studies have associated other genes in the family, such as GTF3B and GTF3C2 are still lacking development.

The functions and networks of GTF3 and its gene enrichment pathways were explored using MetaCore, a high-quality biological platform including integrated pathway and network analyses for multi-omics data. To confirm the prognostic statuses of GTF3 family genes, we conducted integrative data analyses based on the PrognoScan database to explore the relevance of transcript levels of various genes in clinical patients. In this study, functions of GTF3 family members in CRC in terms of novel downstream-regulated networks were systematically examined and discussed for the first time.

## 2. Materials and Methods

### 2.1. Oncomine Analysis and GEPIA Datasets

Oncomine is an online database that provides information on microarray cancer data. ONCOMINE (https://www.oncomine.org/, accessed on 25 February 2021) and GEPIA (http://gepia.cancer-pku.cn/, accessed on 25 February 2021) cancer databases also provide details on gene expression in cancer and normal samples [19,25]. The ONCOMINE and GEPIA analyses in this study were used to determine the expression levels of individual members of the GTF3 family in CRC. Statistical testing was performed using Student’s *t*-test comparisons. The *p*-value was used to make decisions on differences in the gene expressions of GTF3 family members between normal controls and CRC samples. This study used the parameter threshold *p*-value of <0.05, multiple of change of 2, and top 10% gene ranking as we previously described [26,27,28,29,30].

### 2.2. Cancer Cell Line Encyclopedia (CCLE) Analysis

Over 1100 cell lines representing 37 cancer types were explored in the CCLE database (https://portals.broadinstitute.org/ccle, accessed on 25 February 2021), which provides extensive genomic information, computational analyses, and visualization [31]. This study used the CCLE dataset to examine mRNA expression levels to further verify the participation of these GTF3 family members in cancer cell lines. Expression profile values were log-transformed and then visualized by a heatmap.

### 2.3. Differentially Expressed GTF3 Genes: Prognostic Significance and Expression

To determine the prognostic roles of mRNA members of GTF3 family genes in CRC, this study used the PrognoScan (http://dna00.bio.kyutech.ac.jp/PrognoScan/, accessed on 25 February 2021) and HPA databases (www.proteinatlas.org, accessed on 25 February 2021) [32]. The PrognoScan database was used to generate survival plots, with a *p*-value threshold of 0.05 [33]. The HPA database provides a wealth of information on sequences, pathology, expressions, and distributions in various cancer tissues. The first version of this database contained more than 400,000 high-resolution images corresponding to more than 700 antibodies to human proteins [34]. This study analyzed the differential status of protein expressions and localization of select members of GTF3 family genes in CRC tissue using this database.

### 2.4. Genomic Alterations Analysis

The c-BioPortal (https://www.cbioportal.org/, accessed on 28 February 2021) facilitates the exploration of multidimensional cancer genomic data by enabling the visualization and cross-gene analyses of samples, data types, and changes in mRNAs and microRNAs [35,36]. Furthermore, altered gene functions can also occur due to gene mutations [37]. Therefore, we analyzed these genomic changes in GTF3 family members that were differently expressed in CRC.

### 2.5. Functional Enrichment Analysis

We used a pathway enrichment analysis to advance our research, which is generally used to identify cancer risk pathways and describe tumorigenesis processes [38]. This study integrated a cohort profile dataset to illustrate the potential of key candidate genes and pathways in CRC as we previously described [39,40,41,42,43]. Briefly, expression profiles of the GSE17536, GSE17537, and TCGA datasets were integrated and analyzed in depth. The first step was to collect GTF3 family gene expressions in TCGA data using Venny vers. 2.1 (https://bioinfogp.cnb.csic.es/tools/venny/, accessed on 28 February 2021). After that, a Cytoscape study was conducted using the shared gene list. Cytoscape (http://www.cytoscape.org/, accessed on 30 February 2021) [44] is used to visualize networks with expression profiles and other molecules. Furthermore, MetaCore software is used to identify the functions and pathways of altered genes, determine biological processes, disease biomarkers, tissues, colorectal neoplasms, and signaling and regulation of regulated pathways. The results of the enrichment analysis provided a pathway with *p*-value adjustment, and the log transformation was uploaded with a threshold *p*-value of <0.05.

## 3. Results

### 3.1. Expression Pattern of GTF3 Family Genes in CRC

**Figure 1:**
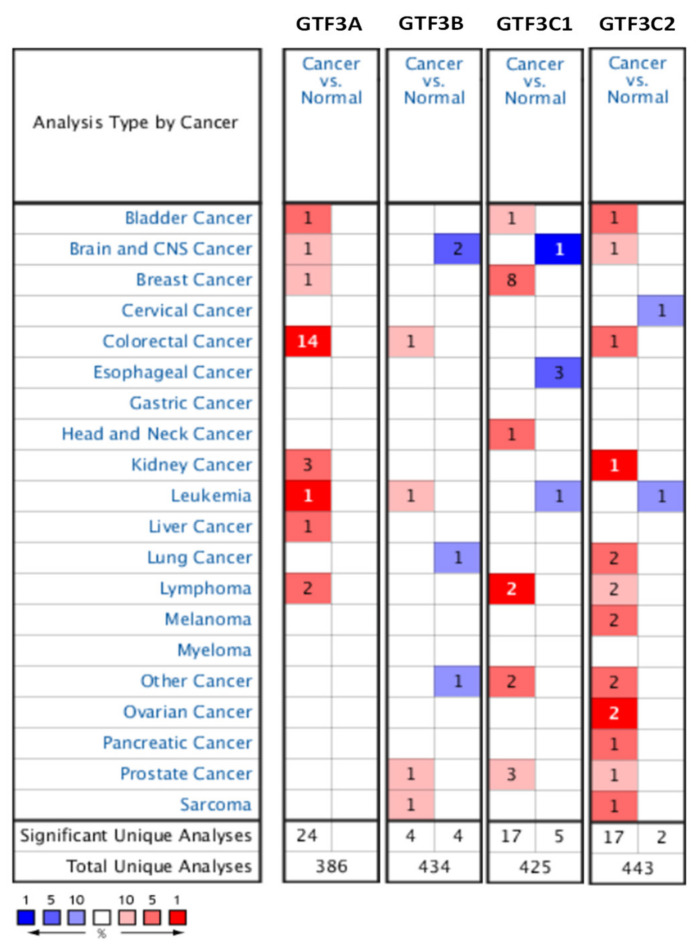
GTF3 gene expression patterns in different cancers (ONCOMINE). The expressions of GTF3 family genes in cancer patients were compared to that of normal patients. A reduction in a gene’s rank percentile is represented by the color gradient. The thresholds applied were a *p*-value of <0.05, a multiple of change of two-fold, and a percentile gene rank of <10% in cancer versus normal cases.

**Figure 2:**
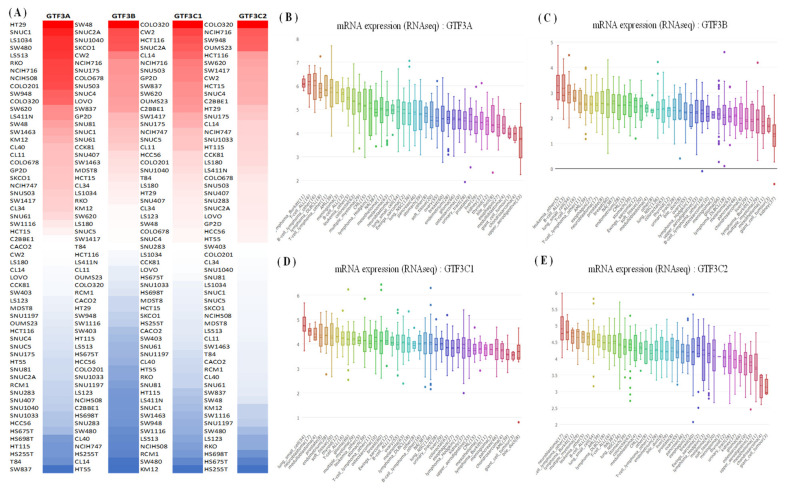
Expressions of members of GTF3 family genes in colorectal cancer cell lines in the CCLE platform. (**A**) The CCLE database was used to plot a heatmap for all rows representing colorectal cancer cells based on expression levels of GTF3 family genes. GTF3 expression levels varied among colorectal cancer cell lines. Red indicates overexpression (top column), and blue indicates under-expression (bottom column) in cancer cells. (**B**) GTF3A expression in colorectal cancer cell lines. (**C**) GTF3B expression in colorectal cancer cell lines. (**D**) GTF3C1 expression in colorectal cancer cell lines. (**E**) GTF3C2 expression in colorectal cancer cell lines.

In the current study, the Oncomine database was used to reveal transcript expressions of GTF3 family genes in cancerous and normal tissues and found that the expressions of GTF3 family genes were associated with many types of cancers. Among all GTF3 members, GTF3A was particularly highly expressed in CRC compared to normal tissues, whereas GTF3B, GTF3C1, and GTF3C2 had low expression levels in other types of cancers such as brain and central nervous system cancers, esophageal cancer, and leukemia (Figure 1). As well as mRNA expression analysis, we also expanded and explored the expression levels of members of the GTF3 family in various cell lines using the Cancer Cell Line Encyclopedia (CCLE) database (Figure 2). The results showed that CRC cell lines, such as HT29, SW48, and COLO320, exhibited significantly high expression levels of GTF3A, GTF3B, GTF3C1, and GTF3C2 (Figure 2A–E).

**Figure 3:**
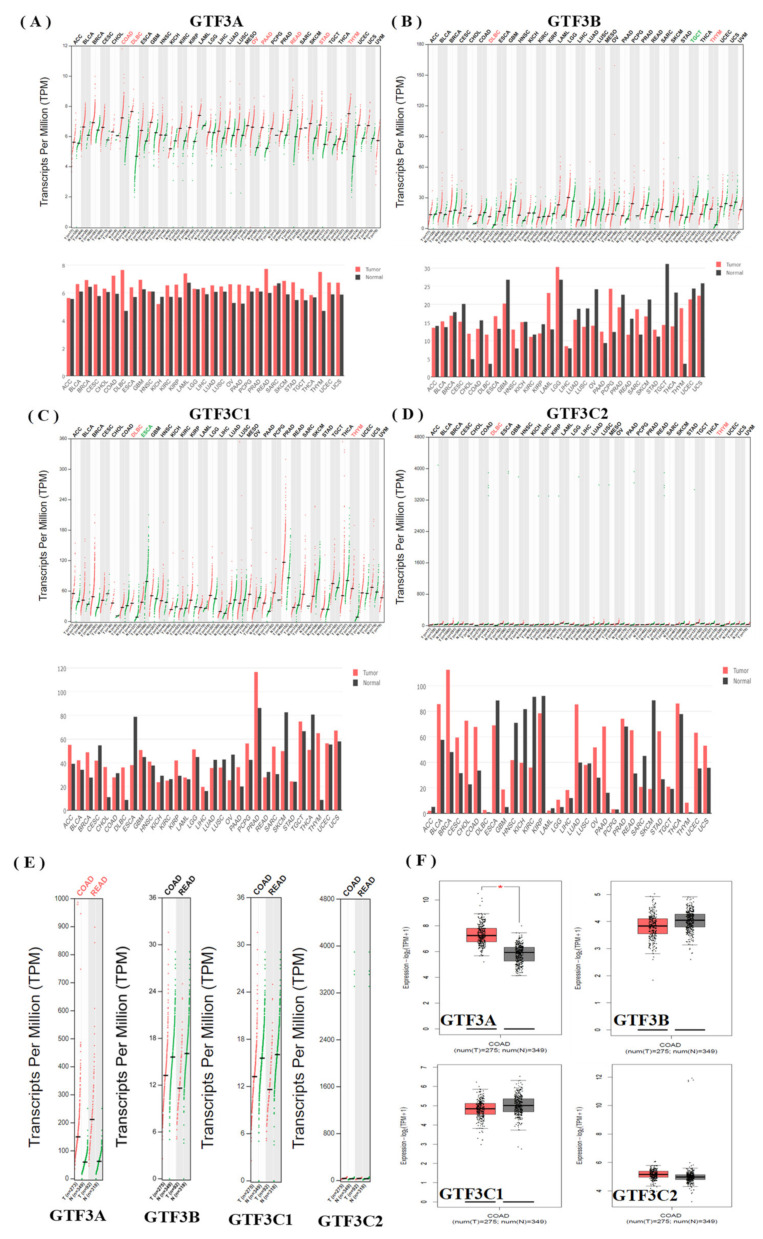
GTF3 expressions in colorectal cancer (GEPIA). (**A**–**D**) Expressions of member genes of the GTF3 family in various types of cancer. (**E**,**F**) Expressions of member genes of the GTF3 family in colorectal cancer. The red bar and box plots show tumor expression while green/black represent normal tissues.

Further analysis identified an association between GTF3 messenger (m)RNA levels in CRC patients. We used GEPIA platform to compare GTF3 mRNA expressions in CRC and normal tissues (Figure 3). Based on GEPIA analysis, the expression level of GTF3A in CRC was higher in tumor tissue relative to normal tissues. In contrast, the expression levels of GTF3B, GTF3C1, and GTF3C2 in CRC were lower in tumor tissue compared to normal tissues (Figure 3A–F).

### 3.2. Prognostic Values and Protein Expressions of GTF3 Family Genes in CRC

**Figure 4:**
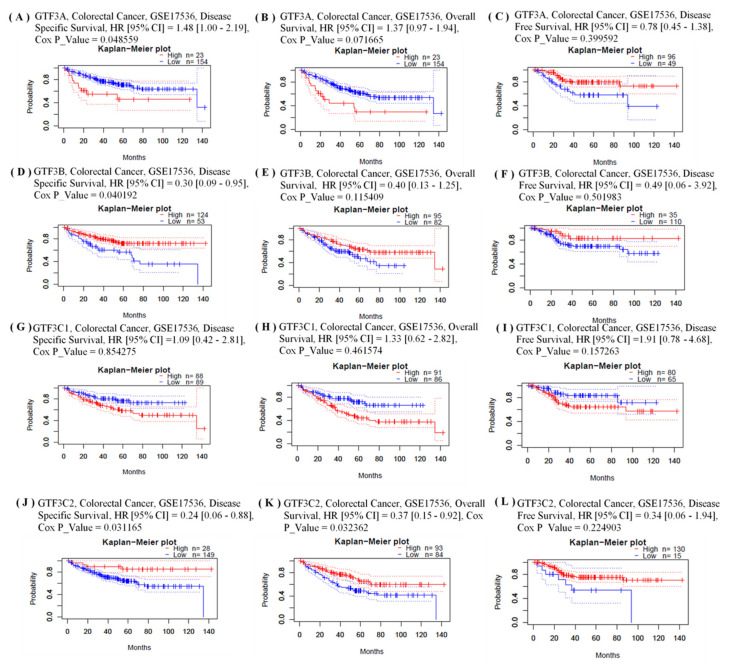
Kaplan–Meier survival curve comparing high and low expressions of genes of members of the GTF3 family in colorectal cancer using the PrognoScan database with GSE17536 cohort data (n = 177). The Kaplan–Meier survival curve shows the correlation of disease-specific survival (DSS), overall survival (OS), and disease-free survival (DFS) of colorectal cancer patients with high and low expression levels of GTF3 family members.

**Figure 5:**
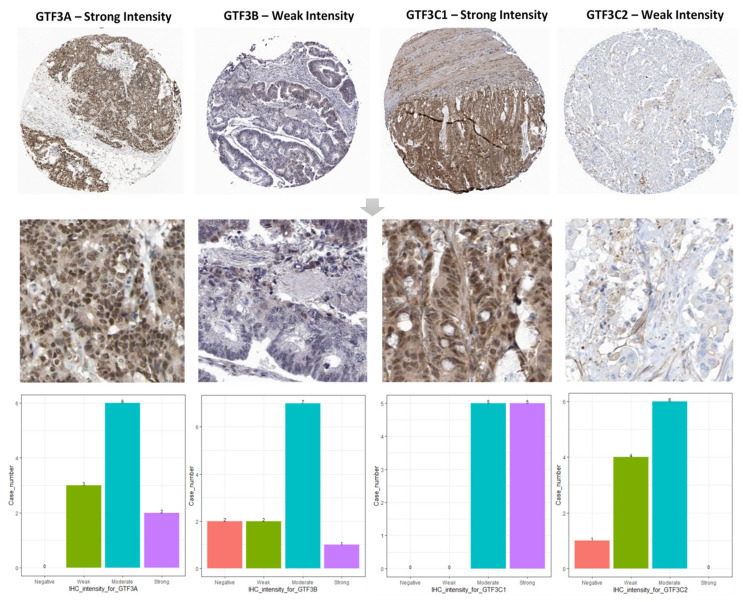
Protein expression levels of members of the GTF3 family in all clinical colorectal cancer specimens from the Human Protein Atlas. GTF3A and GTF3C1 had strong positive protein expressions in colorectal cancer, whereas GTF3B and GTF3C2 had low protein expressions in colorectal cancer.

We investigated whether expressions of GTF3 family genes were correlated with prognoses in CRC patients. The impacts of the expressions of members of this family of genes on survival rates were evaluated using the PrognoScan database [33,45]. An analysis of cohort data according to accession number GSE17536 covering 177 CRC samples showed that GTF3A expression significantly influenced the poor prognosis of CRC patients (disease-specific survival (DSS), hazard ratio (HR) (95% confidence interval [CI]) = 1.48 (1.00–2.19), Cox *p*-value = 0.049), as well as GTF3B (DSS, HR (95% CI) = 0.30 (0.09–0.95), Cox *p*-value = 0.040), GTFC1 expression insignificantly influenced the poor prognosis of CRC patients and GTF3C2 (DSS, HR (95% CI) = 0.24 (0.06–0.88), Cox *p*-value = 0.031; overall survival (OS), HR (95% CI) = 0.37 (0.15–0.92), Cox *p*-value = 0.032) (Figure 4A–L). We next examined in situ expressions of GTF3 family genes at the protein level using immunohistochemical (IHC) data in The Human Protein Atlas (HPA) database. IHC was used to explore the protein levels of GTF3 family members in CRC tissues. We found that GTF3A and GTF3C1 proteins were more highly expressed in CRC tissues than in normal tissues (Figure 5).

### 3.3. Genomic Alterations of GTF3 Family Gene Expressions in CRC

**Figure 6:**
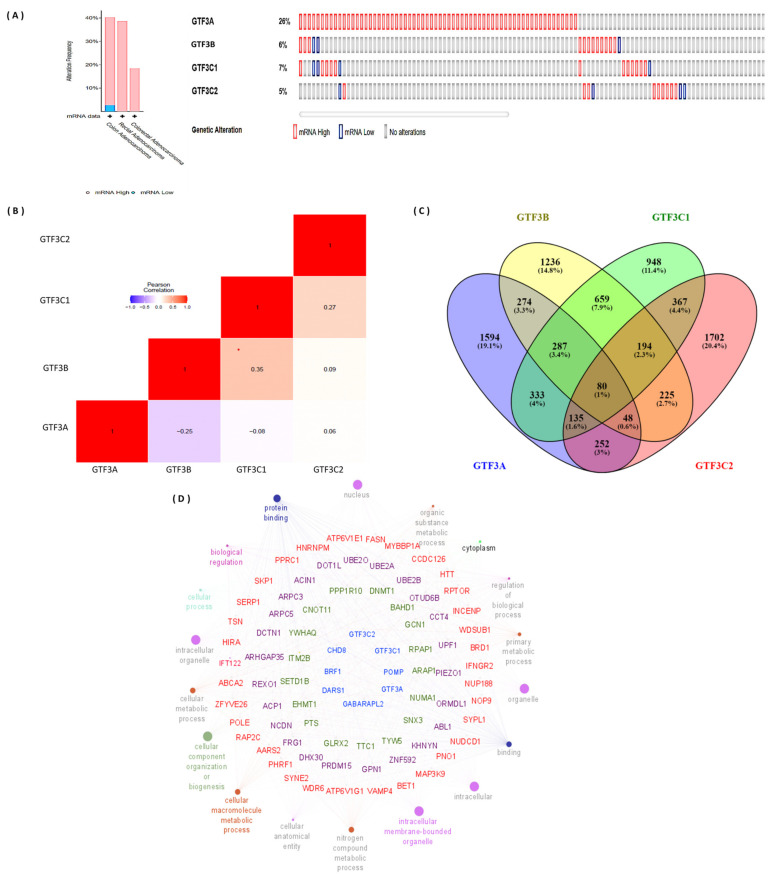
Colorectal cancer has been linked to genomic alterations in differentially expressed GTF3 genes. (**A**) The cBioPortal was used to perform an OncoPrint study of GTF3 family member genes, which showed the degree of gene amplification, deep deletions, and nucleotide substitutions linked to colorectal cancer. (**B**) Correlations between different GTF3s in colorectal cancer (cBioPortal) and insignificant correlations are marked by crosses. (**C**) Venn diagram of the GTF3 family co-expression network in TCGA colorectal cancer databases. The intersection of co-expression genes lists of each gene in the GTF3 family. The approach selected the top 15% of genes from the list of co-expression genes extracted from the TCGA Pancancer dataset. (**D**) Through a Cytoscape analysis, high correlations between GTF3 members and metastasis markers were observed.

Alterations in gene expressions can occur as a result of amplification, deletion of genes, or irregular transcription regulation. Furthermore, altered gene functions can also occur due to gene mutations. Therefore, we analyzed these genomic changes of the GTF3 gene family that were differentially expressed using the cBioPortal (Figure 6) and found that genetic alteration rates of GTF3 family genes were in the order of GTF3A (26%), BRF1/GTF3B (6%), GTF3C1 (7%), and GTF3C2 (5%) (Figure 6A). We also calculated mRNA expression correlations among GTF3 gene family members and found that GTF3A was negatively correlated with GTF3B, GTF3C1, and GTF3C2; GTF3B was positively correlated with GTF3C1 and GTF3C2; and GTF3C1 was positively correlated with GTF3C2 (Figure 6B).

### 3.4. Pathway Enrichment Analysis

We then constructed a network of gene interactions, Gene Ontology (GO) biological processes, and Kyoto Encyclopedia of Genes and Genomes (KEGG) enrichment pathway analyses using 80 shared genes by Venny version 2.1.0, and then used this as an input for ClueGO/CluePedia packages in cytoscape software package (Figure 6C). Statistical options for the ClueGO/CluePedia enrichment analysis were set based on a hypergeometric test that is two-sided with *p* ≤ 0.05, the Benjamini–Hochberg correction, and a kappa score of ≥2 as the primary criteria. Through Cytoscape analysis (GlueGO and CluePedia), we found that members of the GTF3 gene family have a high correlation with metastatic markers such as ABL1 [46,47], WDR6 [48], ARAP1-AS1 [49,50], DNMT1 [51,52,53,54,55], FASN [56,57] (Figure 6D).

### 3.5. GTF Coexpressed Genes and Regulated Networks in TCGA Database

**Figure 7:**
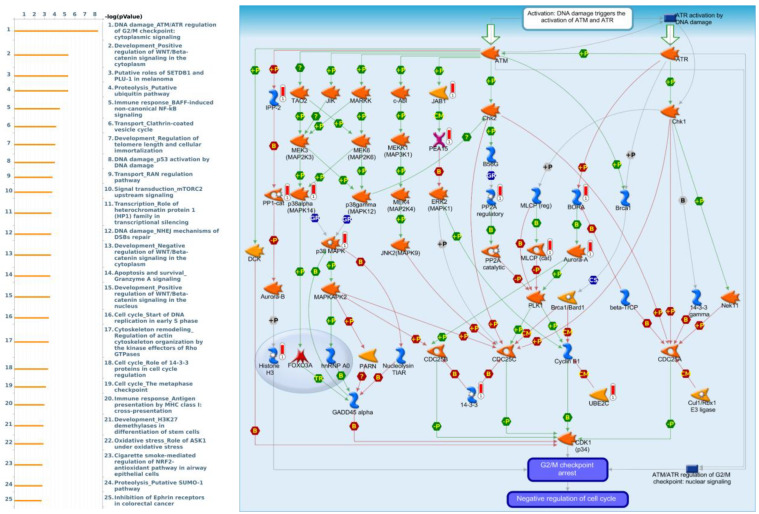
GTF3A differentially expressed genes pathway developed by MetaCore. Experimental data from TCGA were linked to and visualized on pathway maps as thermometer-like figures. To study the gene networks and signaling pathways that could be affected by the chosen genes, we exported genes expressed with GTF3A and then uploaded them to the MetaCore platform for a path analysis. The MetaCore pathway analysis indicated that the “DNA damage_ATM/ATR regulation of G2M checkpoint cytoplasmic signaling”-related pathway was correlated with colorectal cancer development.

To understand how differentially expressed gene (DEG) lists are related to downstream GTF-regulated networks in different biological processes and diseases, we performed an enrichment analysis using MetaCore software. By uploading GTF3A coexpressed genes from the TCGA-CRC dataset into the MetaCore, we revealed that cell cycle-related pathways and networks including “DNA damage_ATM/ATR regulation of G_2_/M checkpoint: cytoplasmic signaling”, “Development_Positive regulation of WNT/Beta-catenin signaling in the cytoplasm”, “Putative roles of SETDB1 and PLU-1 in melanoma”, “Proteolysis_Putative ubiquitin pathway”, and “Immune response_BAFF-induced non-canonical NF-κB signaling” (Figure 7).

**Figure 8:**
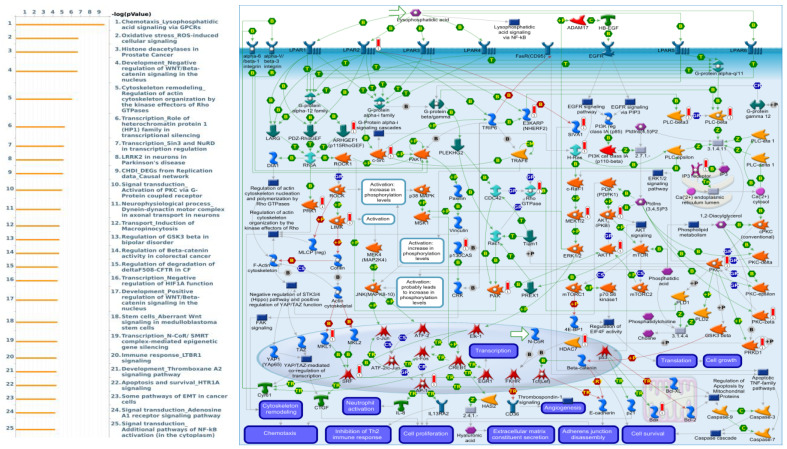
GTF3B differentially expressed genes pathway developed by MetaCore. Experimental data from TCGA were linked to and visualized on pathway maps as thermometer-like figures. To study the gene networks and signaling pathways that could be affected by the chosen genes, we exported genes expressed with GTF3B and then uploaded them to the MetaCore platform for a path analysis. The MetaCore pathway analysis indicated that the “chemotaxis lysophosphatidic acid signaling via GPCRs”-related pathway was correlated with colorectal cancer development.

GTF3B co-expressed genes from the TCGA-CRC database were correlated with “Chemotaxis_Lysophosphatidic acid signaling via GPCRs”, “Oxidative stress_ROS-induced cellular signaling”, “Histone deacetylases in prostate cancer”, “Development_Negative regulation of WNT/Beta-catenin signaling in the nucleus”, and “Cytoskeleton remodeling_Regulation of actin cytoskeleton organization by the kinase effectors of Rho GTPases” (Figure 8).

**Figure 9:**
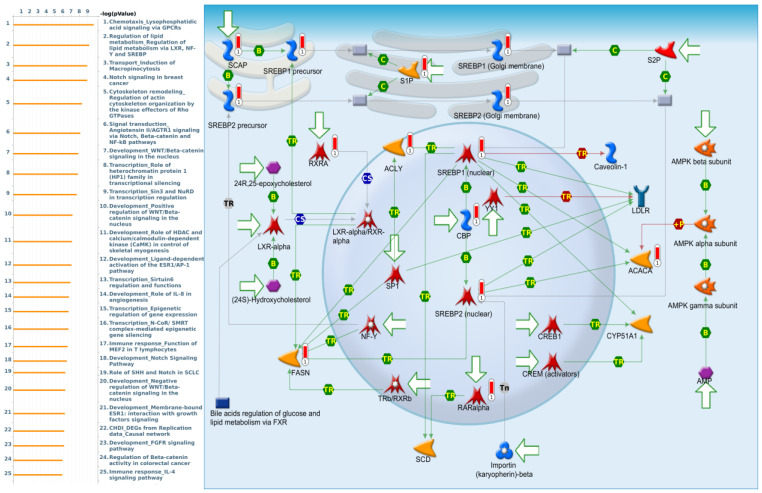
GTF3C1 differentially expressed genes pathway developed by MetaCore. Experimental data from TCGA were linked to and visualized on pathway maps as thermometer-like figures. To study the gene networks and signaling pathways that could be affected by the chosen genes, we exported genes expressed with GTF3C1 and then uploaded them to the MetaCore platform for a path analysis. The MetaCore pathway analysis indicated that the “regulation of lipid metabolism_Regulation of lipid metabolism via LXR, NF-Y, and SREBP”-related pathway was correlated with colorectal cancer development.

GTF3C1 co-expressed genes from the TCGA-CRC dataset were correlated with “Chemotaxis_Lysophosphatidic acid signaling via GPCRs”, “Regulation of lipid metabolism_Regulation of lipid metabolism via LXR, NF-Y and SREBP”, “Transport_Induction of Macropinocytosis”, “Notch signaling in breast cancer”, and “Cytoskeleton remodeling_Regulation of actin cytoskeleton organization by the kinase effectors of Rho GTPases” (Figure 9).

**Figure 10:**
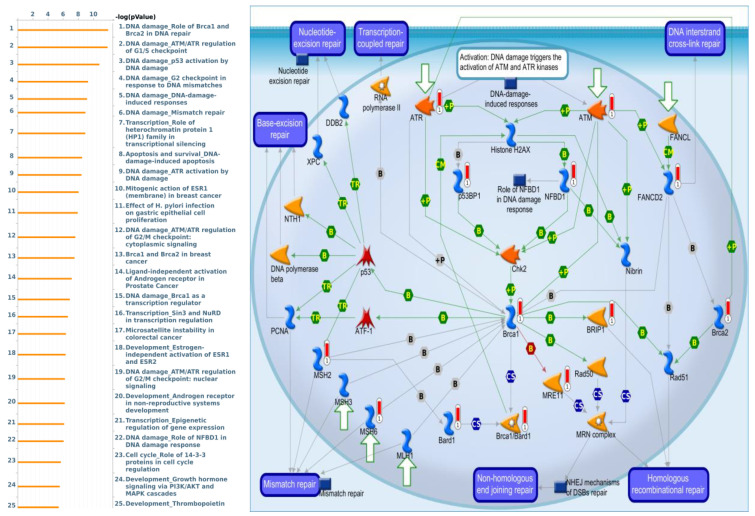
GTF3C1 differentially expressed genes pathway developed by MetaCore. Experimental data from TCGA were linked to and visualized on pathway maps as thermometer-like figures. To study the gene networks and signaling pathways that could be affected by the chosen genes, we exported genes expressed with GTF3C2 and then uploaded them to the MetaCore platform for a path analysis. The MetaCore pathway analysis indicated that the “DNA damage_Role of Brca1 and Brca2 in DNA repair”-related pathway was correlated with colorectal cancer development.

GTF3C2 co-expressed genes from the TCGA-CRC dataset were correlated with “DNA damage_Role of Brca1 and Brca2 in DNA repair”, “DNA damage_ATM/ATR regulation of G1/S checkpoint”, “DNA damage_p53 activation by DNA damage”, “DNA damage_G2 checkpoint in response to DNA mismatches”, and “DNA damage_DNA-damage-induced responses” (Figure 10).

## 4. Discussion

In recent decades, CRC research has dramatically advanced. Prognostic biomarkers, therefore, have also been broadly investigated [58,59,60,61]. Novel targets and signal pathways such as HER2 [62], PIK3CA [63], KRAS [64], and DPP7/2 [65] were discovered. These discoveries have definitely contributed to a deeper understanding of the pathogenesis and occurrence of CRC.

GTF3 family members play crucial roles as transcription factors, affecting several important biological pathways. Nevertheless, these GTF3 transcription factor-related pathways have not yet been clearly elucidated in cancers. Exploring the potential of GTF3s could be a novel approach in cancer therapy for CRC treatment. However, basic studies showed substantial discrepancies in different GTF3 family members’ specific roles in CRC biology [66]. In this research, we systemically represented expression profiles of each member of the GTF3 family, specifically related to CRC, in order to reveal that genes of this family had significant differences by comparing mRNA expressions between CRC tissues and normal colon and rectal tissues. By an integrative analysis with GEPIA, the expression level of GTF3A in CRC was higher in CRC tissue relative to normal tissues. In contrast, the expression levels of GTF3B, GTF3C1, and GTF3C2 in CRC were lower in CRC tissue than those in normal tissues. Hence, among genes of this family, these results confirmed that the GTF3A has distinct mRNA expression in CRC and might imply its effect to this disease.

In order to support these findings at the proteomic level, we examined in situ expressions of genes of the GTF3 family at the protein level using IHC data from the HPA database. We found that GTF3A protein was more highly expressed in CRC tissues than in normal tissues. The above results contradict the previous studies on the protective effect of GTF3A [66,67]. A possible explanation for this conflict may be that GTF3A acts differently in different stages of tumor cells. Further investigations of GTF3A are still needed to resolve these discrepancies.

According to our results, only GTF3A could be considered as a prognostic marker in CRC compared to the other GTF3 family members. Therefore, we further focused on the expression of GTF3A with different clinical parameters. Our Kaplan–Meier analysis via the PrognoScan database revealed that GTF3A expression was positively related to DSS and OS; however, it could not be considered a DFS prognosis marker for CRC patients, with an HR of 0.8 and *p*-value of 0.399. Interestingly, increased in situ GTF3A expression at the protein level using IHC data in the HPA database showed that there were distinguishable patterns between weak and robust expressions in nuclei. Another important result in this study is that we have used ClueGO/CluePedia to improve the biological interpretation of a large list of genes. Multiple lists of markers can be analyzed simultaneously to underline their general or specific function. ClueGO/CluePedia analysis revealed that members of the GTF3 gene family have a high correlation with several metastatic markers in colorectal cancer, such as ABL1, which is highly expressed in tissue and CRC cells, whose high expression is associated with tumor stage of CRC patients [46,47,49]; WDR6 being a potential target gene for miR-451a in CRC [48], lncRNA ARAP1 antisense RNA 1 (ARAP1-AS1) promotes the epithelial-mesenchymal transition (EMT) processes in CRC via the Wnt/β-catenin signaling pathway [49,50]; DYRK2 expression downregulated through transcription regulation by DNMT1 was found to increase colorectal cancer cell proliferation [52]. A high serum FASN level is a prognosis marker of late stage colorectal cancer patients [57]. This study has also revealed that there is a high correlation of GTF3A and the pathway “the DNA damage_ATM/ATR regulation of G2/M checkpoint: cytoplasmic signaling” in CRC development [68]. When DNA double-strand breaks, ATM is a DNA damage signaling [69], and the reduction of ATM was found in CRC tumors [70], Plk1 was elevated in carcinomas of the non-small cell lung and other types of tumor, and it’s also play a crutial role in G2/M checkpoint recovery [71]. The high correlation between GTF3B and the pathway “Chemotaxis_Lysophosphatidic acid signaling via GPCRs” was demonstrated to be involved in CRC development in previous studies [72,73]. Lysophosphatidic acid (LPA) is the smallest bioactive lipid that mediates critical responses such as cell proliferation, migration, and cytoskeletal reorganization by interaction with several G protein-coupled receptors (GPCRs) [74]. LPA signaling promotes cancer development and metastasis by modulating cell proliferation, invasion, adhesion, angiogenesis, and survival [75]. LPA has also been linked to the induction of DNA synthesis and colorectal cancer cell migration [76].

In addition, we also found several genes involved in apoptosis resistance/metastasis through the pathway “DNA_ATM damage/ATR regulation of the G2/M checkpoint: cytoplasmic signaling”, as well as Cell Division Cycle (CDC) family genes such as CDC25A, CDC25B, CDC25C, and CCNB1 (Cyclin B1) [77]. CDC25A has a role in apoptosis/metastasis regulator; CDC25A overexpression increases tumorigenesis, and is often observed in various types of cancer [78]. In expansion to DNA damage, hypoxia was reported to influence CDC25A expression in colon cancer cells [79]. CDC25B was recognized as a target of miRNA-148a which may regulate pancreatic ductal adenocarcinoma development [80]. In vulvar squamous cell carcinoma, overexpression of CDC25B, CDC25C, and phosphor-CDC25C is linked to malignant features and aggressive cancer phenotypes [81]. CCNB1 inhibition causes apoptotic death in certain colorectal cancer cells [82]. Furthermore, CCNB1, which is activated by Chk1, plays an oncogenic function in colorectal cancer cells and may be useful in the development of new colorectal cancer therapies [82].

## 5. Conclusions

In summary, we explored as well as integrated several databases, and high throughput analysis approach revealed that expressions of GTF3 family members play important roles in malignancy development. These result provide useful evidence for prospective research of CRC associations with GTF3 family genes, and these data also suggested that GTF3A might be a potential prognostic biomarker for CRC, although more investigations are needed to determine comprehensively the role of GTF3A in CRC for further translational research.

## Data Availability

Data are available from the corresponding author on reasonable request.
